# The Himalayan Onion (*Allium wallichii* Kunth) Harbors Unique Spatially Organized Bacterial Communities

**DOI:** 10.1007/s00248-021-01728-5

**Published:** 2021-03-16

**Authors:** Xiaoyulong Chen, Lisa Krug, Maofa Yang, Gabriele Berg, Tomislav Cernava

**Affiliations:** 1 Key Laboratory of Green Pesticide and Agricultural Bioengineering, Ministry of Education, Guizhou University, Guiyang, 550025 China; 2 College of Tobacco Science, Guizhou University, Guiyang, 550025 China; 3 Key Laboratory of Agricultural Microbiology, Guizhou University, Guiyang, 550025 China; 4 College of Science, Tibet University, Lhasa, 850012 China; 5 Institute of Environmental Biotechnology, Graz University of Technology, 8010 Graz, Austria

**Keywords:** Plant microbiome, Bacterial communities, Endophytic bacteria, Rhizosphere, Phyllosphere

## Abstract

Plant-associated microorganisms are known to contribute with various beneficial functions to the health and productivity of their hosts, yet the microbiome of most plants remains unexplored. This especially applies to wild relatives of cultivated plants, which might harbor beneficial microorganisms that were lost during intensive breeding. We studied bacterial communities of the Himalayan onion (*Allium wallichii* Kunth), a wild relative of onion native to mountains in East Asia. The bacterial community structure was assessed in different plant microhabitats (rhizosphere, endosphere, anthosphere) by sequencing of 16S rRNA gene fragment amplicons. Targeted bioinformatic analyses were implemented in order to identify unique features in each habitat and to map the overall community in the first representative of the Amaryllidaceae plant family. The highest bacterial diversity was found for bulk soil (Shannon index, *H*′ 9.3) at the high-altitude sampling location. It was followed by the plant rhizosphere (*H*′ 8.9) while communities colonizing flowers (*H*′ 6.1) and the endosphere (*H*′ 6.5 and 5.6) where less diverse. Interestingly, we observed a non-significant rhizosphere effect. Another specificity of the microbiome was its high evenness in taxonomic distribution, which was so far not observed in plant microbiomes. *Pseudomonas* was identified among additional 10 bacterial genera as a plant-specific signature. The first insights into the microbiome of a plant in the widespread *Allium* genus will facilitate upcoming comparisons with its domesticated relatives while additionally providing a detailed microbiome mapping of the plant’s microhabitats to facilitate bioresource mining.

**Supplementary Information:**

The online version contains supplementary material available at 10.1007/s00248-021-01728-5.

## Introduction

Various members of the Amaryllidaceae plant family find application in traditional as well as modern cuisine. They are often characterized by rich secondary metabolite profiles, which can include sulfur-containing compounds with a characteristic smell [1]. Globally, the most relevant members are constituted by various cultivars of the common onion (*Allium cepa* L.) and garlic (*Allium sativum* L.). The direct ancestor of onions is likely extinct; however, most of the recent wild relatives originate from Asia. Similar to their cultivated relatives, distinct wild species of the Amaryllidaceae also find application as ingredients of traditional dishes as well as sources of bioactive compounds in phytomedicine [2–4]. This includes the Himalayan onion *Allium wallichii* Kunth, a perennial herb with a single, conical, and short bulb, which is native to India, Nepal, Bhutan, Myanmar, and Southwestern China (Guangxi, Guizhou, Hunan, Sichuan, Tibet, and Yunnan provinces). The anatomy of the plant is reminiscent of the spring onion (*Allium fistulosum* L.), and it often occurs in natural monocultures. Another characteristic of the plant is that it grows at elevations of 2300 to 4800 m [5]. Jiucaiping Mountain, with an altitude of 2900 m above sea level, is the highest point of Guizhou Province, China, and harbors the largest known habitat (approx. 700 ha) of wild *A. wallichii* in the Yunnan-Guizhou Plateau [6]. The mountain is known for its unique landscape during flowering season of *A. wallichii* in August and September. The extracts from the Himalayan onion were shown to be promising candidates for medicinal applications due to anti-cancer activity and low cytotoxicity [7]. In addition, the extracts exerted anti-microbial activity against known pathogens including *Bacillus cereus*, *Escherichia coli*, and *Pseudomonas aeruginosa* [8]. However, so far, nothing is known about the microbiome found in natural vegetation predominantly covered by *A. wallichii* and microbial communities colonizing the plant itself.

During the last years, the plant microbiota was shown to play a crucial role for host health, protection against phytopathogens, and resistance towards abiotic stresses [9, 10]. Plants were shown to acquire microorganisms from the surrounding environment, but can also equip their seeds with beneficial endophytes [11, 12]. In the plant rhizosphere, rhizobacteria and mycorrhizal fungi can assist plants against drought stress and contribute to the nutrition by nitrogen fixation [13] as well as increase the availability of phosphorus [14] and potassium [15]. Additionally, beneficial microbes that naturally occur in association with distinct plants can be used as a resource for novel biocontrol and plant growth promotion applications in other vegetation systems [16, 17]. In addition to commonly exploited microorganisms from soil, the plant rhizosphere and endorhiza, microorganism from aboveground tissues, including stem and flower (anthosphere), are also promising candidates for biotechnological applications [18, 19]. Therefore, in order to provide a complete map of a plant’s microbial community, it is important to include all major tissue types in the analysis. Bacteria show specificity not only for plant tissues but also for plant genotypes [20, 21]. In this context, it was shown that plants with bioactive secondary metabolites usually harbor highly specific microbial communities that are shaped by those substances [22–24]. Less is known about the bacterial component within the microbiome of the Amaryllidaceae plant family, while its mycorrhiza was already intensively studied [25]. Moreover, the microbiome of stored onions (*Allium cepa*) was recently explored by Yurgel and colleagues [26]; they identified *Pseudomonas* as a biomarker for healthy onions.

Our objective was to analyze the microbiome of *Allium wallichii* grown in their native environment to provide a first basis to understand and manage the microbiome of our oldest and still widely used crops: onion and garlic. Due to the unique karst landscape and large-scale natural monoculture, we hypothesized that *Allium wallichii* plants growing on the Jiucaiping Mountain, which is a central part of the Yunnan-Guizhou Plateau, are colonized by uniquely composed, spatially organized bacterial communities in its different tissues. *Pseudomonas*, the biomarker for healthy onions [26], was expected to be a prevalent colonizer of their wild relative. Therefore, we explored the microbiome of *Allium wallichii* by amplicon-based 16S rRNA gene sequencing, in order to make it available as a novel bioresource for upcoming biotechnological applications.

## Material and Methods

### Sample Collection and Total Community DNA Extraction

All samples were obtained from a large, natural *A. wallichii* monoculture growing on Jiucaiping Mountain in Guizhou Province (China; 26° 59′ 42″ N, 104° 45′ 36″ E). All plants were unearthed during the flowering stage and stored on ice in sterile zip bags until their arrival in the laboratory. Soil samples that were used for complementary analyses were samples in a radial distance of 20–30 cm from the plants. First, the top soil was removed, then root-free material was obtained from a depth of 10 cm and immediately manually homogenized inside sterile zip bags to remove macro-aggregates. For soil samples, four replicates were obtained, while for the rhizosphere samples and each plant tissue type, six replicates were obtained, respectively. The plant samples were further processed in the laboratory by separating different plant tissues with sterile scalpels. Rhizosphere samples were obtained by washing adhering soil from roots in 20 ml of 0.85% NaCl at 120 rpm for 10 min. The suspension was aliquoted into 2-ml reaction tubes and centrifuged at 14,000*×g* for 20 min to obtain pellets for further processing. Samples of the endorhiza and the pedicule endosphere were obtained by surface sterilizing 3–5-cm-long segments of each sample type. The segments were first submerged in 4% sodium hypochlorite (NaClO) solution and placed on a shaker (120 rpm) for 5 min. Subsequently, the segments were rinsed with sterile ddH_2_O and then washed in sterile reaction tubes with sterile ddH_2_O on a shaker (120 rpm) for 5 min. The washing step was repeated for two times in order to remove residual NaClO from the samples. Flowers were directly removed from the plant under sterile condition and subjected to homogenization without further treatments. Subsequently, the plant samples were treated with an automated tissue homogenizer (BBI Life Sciences; Shanghai, China). The homogenates as well as the soil and rhizosphere samples were transferred into extraction tubes under sterile conditions. The total community DNA was extracted from all samples with the FastDNA SPIN Kit for Soil (MP Biomedicals, USA) following the manufacturer’s protocol. Subsequently samples were analyzed with NanoDrop (Thermo Fisher Scientific, USA) to confirm the DNA recovery.

### Amplification of 16S rRNA Gene Fragments

The total community DNA extracts from six replicates per sample type were used for PCR-based amplifications with the primers 515f (5′ GTGYCAGCMGCCGCGGTAA) and 806r (5′ GGACTACHVGGGTWTCTAAT) according to the Earth Microbiome Project protocol (www.earthmicrobiome.org) [27] with sample-specific barcodes and Illumina sequencing adaptors. In addition, specific peptide nucleic acid (PNA) oligomers were added to the PCR mix to prevent the amplification of mitochondrial (mPNA) or plastidial (pPNA) RNA from eukaryotes [28]. The resulting amplicons were used to construct a next-generation sequencing library suitable for sequencing on an Illumina PE250 platform and processed with a targeted output of ≥ 100,000 reads per sample (Novogene; Beijing, China).

### Bioinformatic Analyses

The data was subjected to a standardized workflow for further quality filtering and dereplication. Paired end reads obtained from an Illumina PE250 instrument (paired-end reads; 250-bp read length) were assigned to samples based on their unique barcode sequence and truncated by removing barcode and primer sequences by the sequencing company (Novogene; Beijing, China). Demultiplexed paired end reads were imported in QIIME 2 (2018.11) and further subjected to quality filtering. By applying the dada2 algorithm, paired-end sequences were denoised and dereplicated and chimeras were removed. Taxonomic analysis of amplicon sequencing variants (ASVs) was based on a Naïve-Bayes classifier that was trained on the SILVA 128 release database with clustering at 99% similarity [29]. The generated feature table (Table S1) was used for all subsequent analyses. After removing mitochondrial, chimeric, and plastid sequences, the 16S rRNA dataset was normalized to 1677 reads per sample to account a variation in the samples reaching from a maximum of 616,087 to a minimum of 1677 reads. Following rarefication of the feature table, alpha and beta diversity analyses were conducted in combination with complementary statistics using QIIME 2 core diversity metrics. Principal coordinate analysis (PCoA) plots were constructed on the basis of an unweighted UniFrac distance matrix that is implemented in QIIME 2. Phylogenetic metrics were generated by aligning representative sequences using the mafft program. After masking the multiple sequence alignment, a phylogenetic tree was generated with FastTree. Statistics were calculated by applying analyses of similarity (ANOSIM) within the QIIME 2 pipeline for identifying significant differences in beta diversity between groups. The alpha diversity was assessed through observed OTU and Shannon diversity indices and statistics calculated by applying Kruskal-Wallis tests including Benjamini/Hochberg FDR adjustment within QIIME 2. Significantly different abundant features were identified by applying the Kruskal-Wallis test including FDR multiple test correction within QIIME 1.9.0. As input for calculations, a non-rarefied feature table excluding mitochondrial and plastid sequences was used. Differences in evenness were calculated using one-way ANOVA including Bonferroni multiple test correction in IBM SPSS (version 25.0; IBM Corporation, NY, USA).

For constructing the OTU network, the feature table was reduced by retaining only features which occur in ≥ 75% of the replicates in each group (further referred to as core microbiota) and with a mean relative abundance of at least 0.1% in the whole dataset. The OTU network was constructed using the QIIME 1.9.0 pipeline (make_otu_network.py) and visualized using Cytoscape version 3.7.0. For generating bar charts, features were collapsed on genus level and a reduced table containing only taxa with a mean relative abundance of at least 0.5% in the whole dataset served as input. Bar charts represent mean relative abundance of each group. A collapsed feature table (genus level) served also as input for the generation of the phylogenetic tree including all taxa with a mean relative abundance of at least 0.05% within the whole dataset. A phylogenetic tree was constructed with extracted *Pseudomonas* ASVs by using MEGA X (version 10.0.4) and the implemented neighbor-joining method with 1000 bootstraps.

### Physicochemical Soil Analyses

Bulk soil was obtained at the same sampling location as *A. wallichii* and collected at a depth of 10 cm, which corresponds to the approximate root depth of the plants. A total of 500-g soil was obtained and characterized in terms of pH level, organic matter fraction, total nitrogen content, total phosphorous content, total potassium, total water-soluble salts, and cation exchange capacity by the service provider United Nation Quality Detection Co., Ltd. (Xi’an, Shaanxi, China).

## Results

### Diversity Analyses

**Fig. 1:**
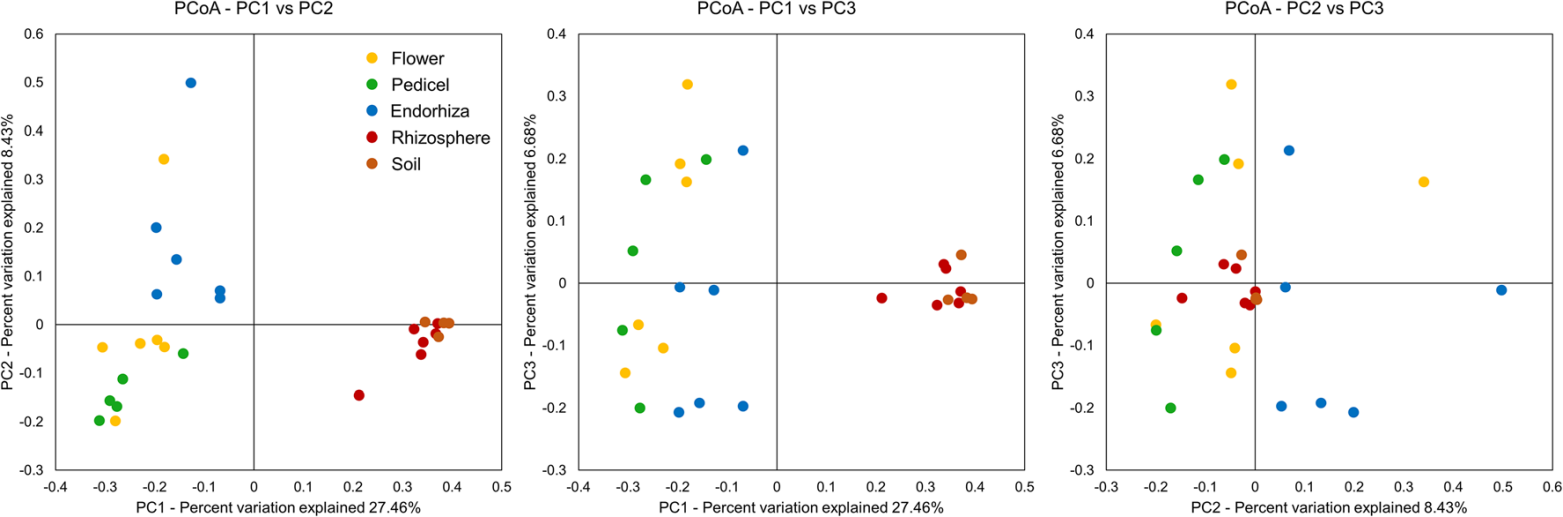
Beta diversity assessment in different plant tissues, the rhizosphere, and surrounding soil. A principal coordinate analysis (PCoA) was conducted to visualize community structure similarity and clustering between different sample types. The percentages of the three dimensions, which explain the highest degree of variance, were included in the visualization. Different colors of the dots indicate distinct sample types in the plant microbiome and the bulk soil. Complementary statistical analyses were used to assess the significance of dissimilarity between samples and summarized in Table S4

**Fig. 2:**
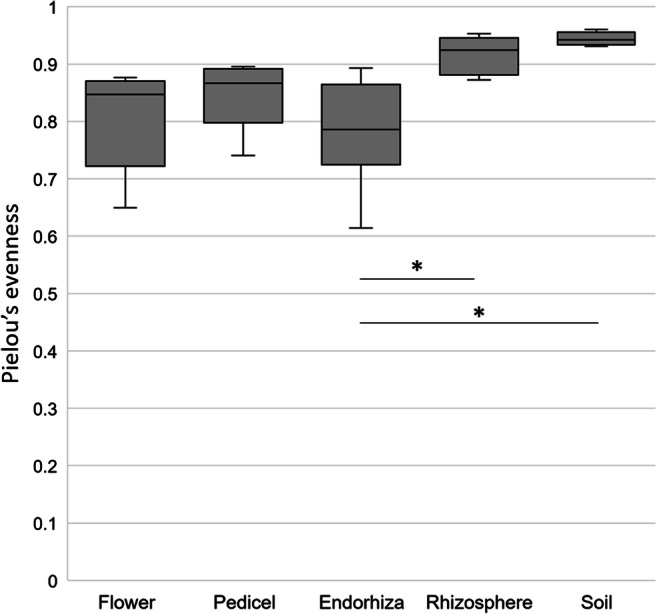
Evenness of the plant-associated bacterial communities and bulk soil. The dataset was subjected to Pielou’s evenness analysis in order to obtain a numeric measure for the evenness that was evident from the community structures across different sample types. Significant differences in evenness were calculated by pairwise comparison using ANOVA including Banferroni multiple test correction and summarized in Table S5

After filtering chimeric, mitochondrial, and plastid sequences, feature table was reduced from a total read count of 8,925,788 to 3,380,349 sequences representing 14,140 features. Highly abundant features that remained without classification at lower taxonomic levels were manually blasted against the NCBI nt_collection; results indicated that several of those features were plastid or chimeric sequences and were therefore excluded prior to further analyses. The filtered feature table contained 3,250,046 sequences representing 13,361 features. Following normalization of the feature table to 1677 reads per sample, 5266 features remained. These features were collapsed on genus level resulting in 992 taxa. Alpha rarefaction analyses revealed the highest Shannon index for soil (*H*′ = 9.34 ± 0.10) followed by the rhizosphere (*H*′ = 8.87 ± 0.46). Shannon diversity indices for flower, pedicel, and endorhiza were calculated as 6.11 ± 0.80, 6.50 ± 0.44, and 5.59 ± 0.93, respectively, whereas no significant differences in alpha diversity were observed within these plant-associated microhabitats (*p* > 0.05). A detailed breakdown of Shannon indices and complementary statistics is shown in Table S2. The same results were obtained when assessing bacterial diversity based on observed OTUs. The highest number of observed OTUs was revealed for soil (950 ± 24) followed by the rhizosphere (822 ± 107). Flower (195 ± 45), pedicel (204 ± 31), and endorhiza (146 ± 49) shared similar numbers of observed OTUs with no significant differences (Figure S1; Table S3). Beta diversity was assessed by calculating the unweighted UniFrac distance matrix, and statistics were calculated using pairwise ANOSIM. Distinct clustering of plant-associated microhabitats (flower, pedicel, endorhiza) and soil-associated habitats (rhizosphere and soil) was observed, reflecting the statistical analyses; within the plant (flower, pedicel, endorhiza), no significant (*p >* 0.05) differences in community compositions were calculated; the same observation was made when differences in beta diversity between rhizosphere and soil were assessed (Fig. 1; Table S4). Community compositions differed significantly when pairwise comparisons were conducted between each plant microhabitat and either soil or the rhizosphere. All samples showed a very high evenness whereas bacterial features are most evenly distributed within the soil (*E* = 0.944 ± 0.010) followed by the rhizosphere (*E* = 0.917 ± 0.030). Significant differences in evenness were found between the endorhiza (*E* = 0.783 ± 0.088) and soil as well as between the endorhiza and the rhizosphere, respectively (Fig. 2; Table S5).

### Bacterial Community Structure in Different Plant Microhabitats

**Fig. 3:**
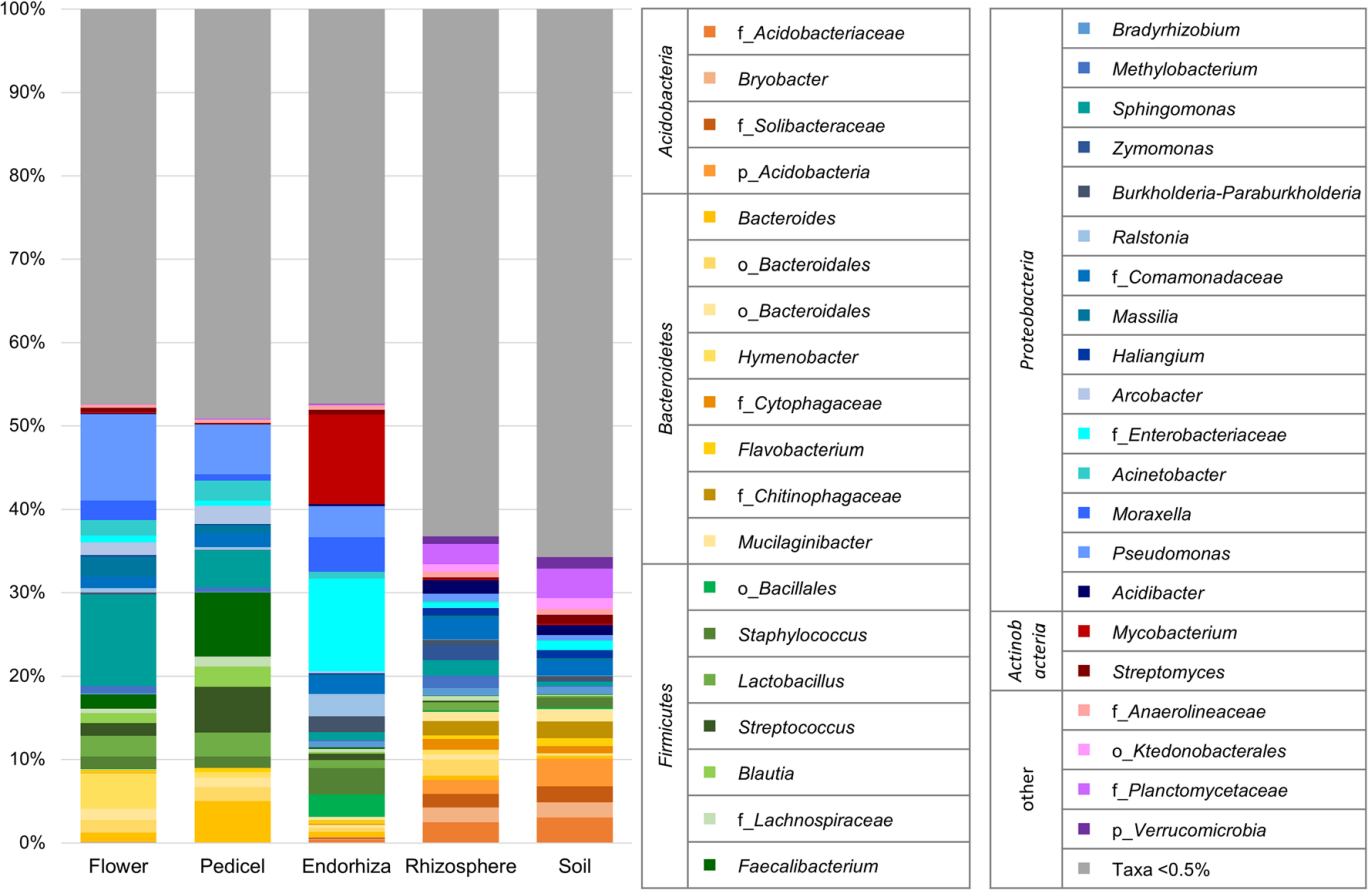
Bacterial community structure in the *A. wallichii* microbiome. The bacterial community structure was visualized up to genus level for each sample type. When genus-level assessment was not possible, the taxonomic information was provided on family, order, or phylum level and indicated with a prefixed letter. Taxonomic assignments at higher levels are provided and clustered in the legend. All taxa with an occurrence of ≥ 0.5% were included in the legend. Taxa with a lower abundance were summarized as “taxa < 0.5%”﻿

Overall, the normalized feature table contained 42 different phyla; among them, *Proteobacteria* were predominating in the rhizosphere (39%) followed by *Bacteroidetes* (14%), *Acidobacteria* (12%), *Actinobacteria* (7%), *Firmicutes* (6%), *Chloroflexi* (6%), *Planctomycetes* (6%), *Verrucomicrobia* (5%), and *Gemmatimonadetes* (1%). Within the soil, the dominating phyla were *Proteobacteria* (28%) followed by *Acidobacteria* (17%) and *Bacteroidetes* (11%), *Actinobacteria* (9%), *Planctomycetes* (8%), *Chloroflexi* (7%), *Verrucomicrobia* (6%), *Firmicutes* (5%), and *Gemmatimonadetes* (1%); with 5% relative abundance, *Archaea* were most abundant in soil while in all other samples, their relative abundance was less than 1%. In the flower, the most abundant bacterial phyla were *Proteobacteria* (51%), followed by *Firmicutes* (20%), *Bacteroidetes* (15%), *Actinobacteria* (7%), *Deinococcus-Thermus* (1%), and *Chloroflexi* (1%). The pedicel was dominated by *Firmicutes* (40%) followed by *Proteobacteria* (33%), *Bacteroidetes* (13%), and *Actinobacteria* (8%). In the endorhiza, the most abundant phyla were *Proteobacteria* (47%) followed by *Firmicutes* (19%), *Actinobacteria* (18%), *Chloroflexi* (2%), and *Acidobacteria* (2%). Phyla with a relative abundance of less than 0.5% were not included in deepening assessments (Fig. 3; Table S6). In order to detect habitat-specific occurrence of distinct bacterial features, a statistical assessment of differential abundance was conducted. Within the plant tissues (flower, pedicel, endorhiza), no significantly different abundant features were identified. The same observation was made when soil and rhizosphere were compared. However, when plant tissue-associated taxa were contrasted to such occurring in the other microhabitats (group A: flower, pedicel, endorhiza; group B: rhizosphere, soil), 3595 of 13,361 features (27%) were significantly different abundant in the compared groups. Of those 3595 features, only 11 were unique for the plant tissues (*Nocardiopsis*, *Hymenobacter*, *Pseudomonas*, *Aureimonas*, *Clostridium*, *Macellibacteroides*, *Bacteroides*, not further classified *Verrucomicrobiaceae*, not further classified *Microbacteriaceae*, *Micrococcus*, *Phascolartobacterium*), while 3369 were unique in the rhizosphere/soil group. In total, 215 features were found in both groups, but showed significantly different abundances therein. Only seven of those 215 features were significantly more abundant in the plant than in the rhizosphere/soil. Those features were assigned to the bacterial genera *Streptococcus*, *Mycobacterium*, *Bacteroides*, *Subdoligranulum*, not further classified *Lachnospiraceae*, and not further classified *Ruminococcaceae*.

### Habitat-Specific Microbial Signatures

**Fig. 4:**
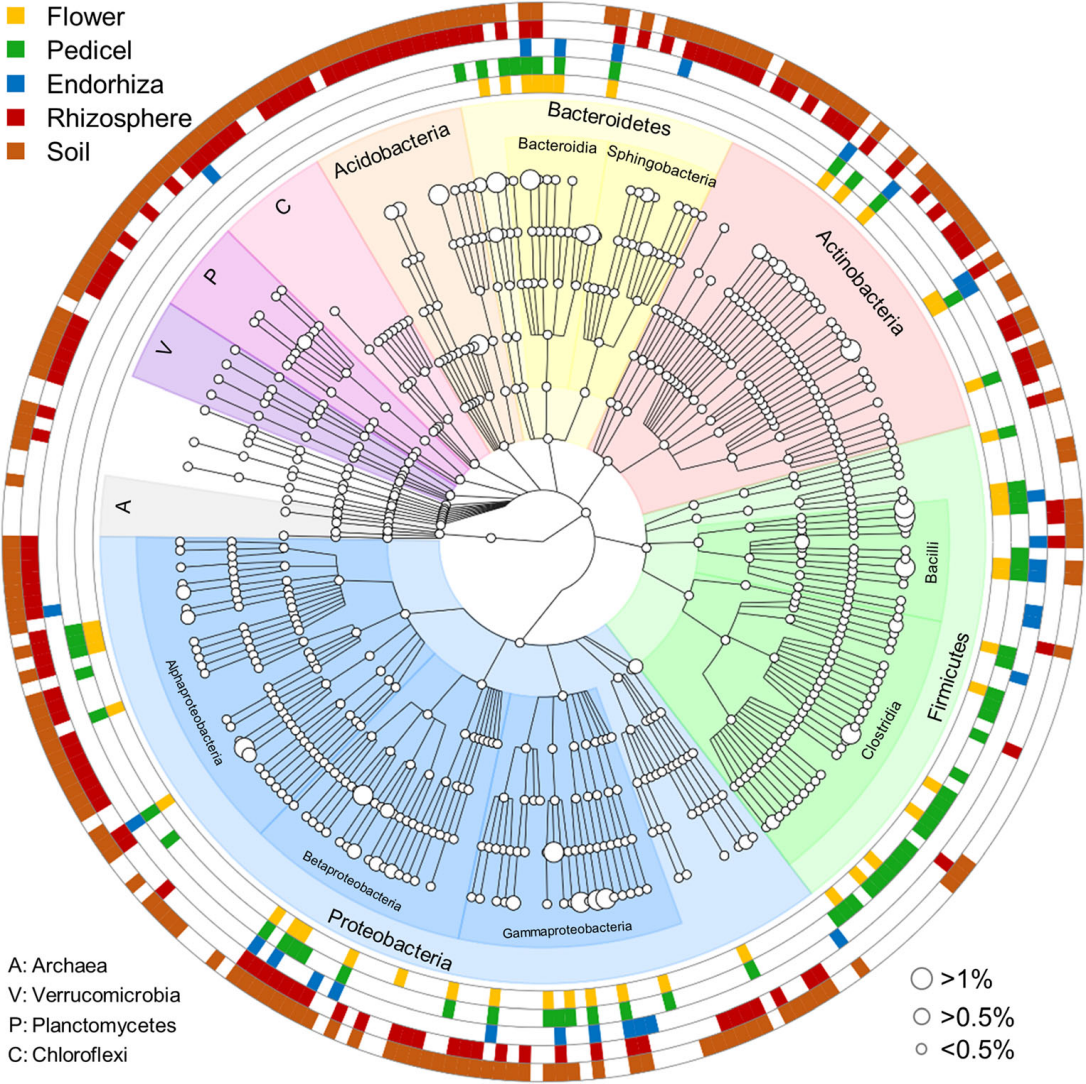
Assessment of common bacterial taxa in different tissues of *A. wallichii*. The tree graph includes taxa with a minimum relative abundance of 0.05% within the whole dataset. All features were collapsed at genus level to reduce the complexity of the visualization. Coloration in the outer rings indicates the occurrence of distinct taxa in the indicated sample types. Node sizes correspond to the mean relative abundance of distinct taxa over the whole dataset; three representative node sizes are provided with the respective percentages

A phylogenetic tree, which was constructed on the basis of prevalent members of the microbiome, included 269 taxa with a mean relative abundance of 0.05% over the whole dataset (Fig. 4). For the flower, the pedicel, and the endorhiza, 44, 62, and 29 bacterial taxa were identified to occur in their respective core microbiota. The rhizosphere harbored 131 core taxa and the soil 177. Overall, only seven bacterial taxa occurred in the core microbiota of all analyzed microhabitats (*Pseudomonas*, *Sphingomonas*, not further classified *Comamonadaceae*, *Bacteroides*, not further classified *Bacteroidales*, *Acinetobacter*, and *Duganella*). Five core taxa were identified to be unique in plant microhabitats (flower, pedicel, endorhiza) including *Streptococcus*, *Nocardiopsis*, *Prevotella*, *Corynebacterium*, and *Propionibacterium*, while soil and rhizosphere shared in total 98 core taxa. Only one unique core taxon was identified for the flower (*Macellibacteroides*) while 19 taxa occurred only in the core microbiota of the pedicel and four taxa were identified as unique core taxa for the endorhiza (not further classified *Planococcaceae*, *Romboutsia*, *Tumebacillus*, *Coxiella*). The soil harbored 40 unique core taxa, while for the rhizosphere, no unique core taxa were identified. Due to the prominent occurrence of *Pseudomonas* in plant-associated microhabitats, a detailed analysis was conducted with all representatives that were recoverable from the amplicon library (Figure S2). A genus-specific phylogenetic tree that was constructed with the recovered *Pseudomonas* ASVs and available reference sequences indicated the presence of potentially novel lineages that showed low sequence similarity to available isolates.

### Assessment of Connectivity Between Plant Microhabitats

**Fig. 5:**
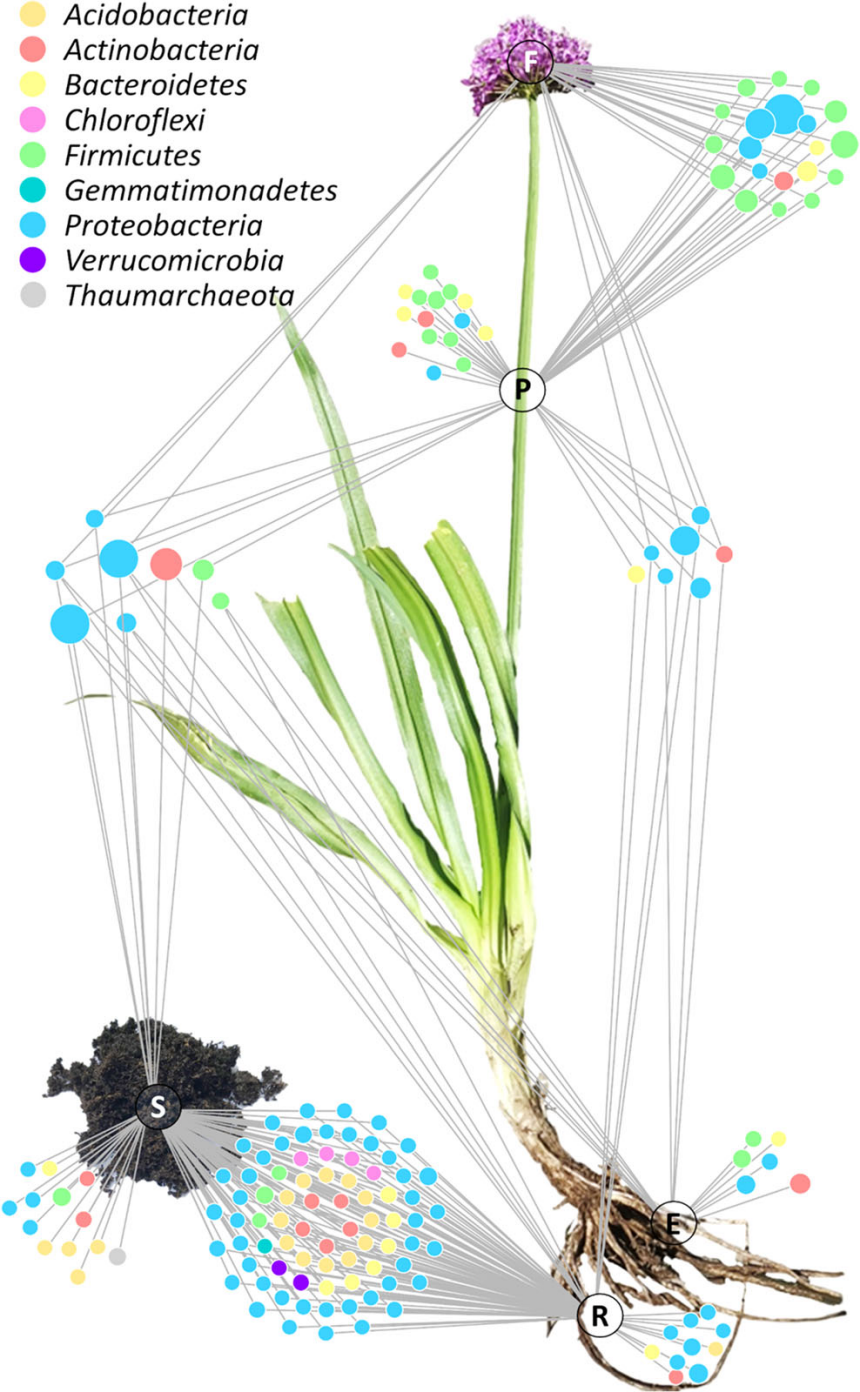
Identification of common bacterial components across plant habitats at feature level. The network was rendered with Cytoscape 3.7.0 in order to identify shared bacterial features in the plant microbiome. The different sample types were mapped on an *A. wallichii* illustration to better illustrate the localization of the analyzed samples in the plant. Edges connect each feature with its origin while multiple edges indicate occurrence in different sample types. Node colors indicate the assignment to distinct bacterial phyla and their size correlates with the abundance of each feature

The connectivity between all analyzed microhabitats was analyzed by constructing a feature-level network. All features were visualized as nodes and connected with edges to the respective microhabitat of the plant where they were detected. In this analysis, which is based on a higher taxonomic resolution than the genus-level profiling, most features were found to be shared between the rhizosphere and the soil (76 features), including 43 features assigned to *Proteobacteria*, 12 features assigned to *Acidobacteria*, six to *Bacteroidetes*, five features assigned to *Actinobacteria*, four to *Chloroflexi*, three to *Firmicutes*, two to *Verrucomicrobia*, and one to *Gemmatimonadetes*. The second highest number of features (20) was shared between the flower and the pedicel with 12 features assigned to *Firmicutes*, five features to *Proteobacteria*, two to *Bacteroidetes,* and one to *Actinobacteria*. While the flower harbored no unique features, the most unique features (15) were identified for the pedicel including seven features assigned to *Firmicutes*, four to *Bacteroidetes,* two to *Actinobacteria*, and two assigned to *Proteobacteria.* The endorhiza, the rhizosphere, and the soil harbored six, 10, and 13 unique features, respectively. Only two features were shared by all microhabitats; they were assigned to *Pseudomonas* and *Duganella*. In addition, it was found that one feature assigned to *Bacillales* was shared between the endorhiza and the rhizosphere (Fig. 5).

### Soil Physicochemical Parameters

The sampled soil from the *A. wallichii* field on Jiucaiping Mountain is typical clay loam. It had a close to neutral pH of 6.81, a cation exchange capacity of 12.2 cmol/kg, and a medium salinity with 8.5 g/kg water-soluble salts. In terms of plant nutrients, the total nitrogen content was 1.31%, the organic matter fraction 1.48%, the total phosphorus 601 mg/kg, and the total potassium concentration 114.24 mg/kg.

## Discussion

The present study provides a detailed profiling of a native plant to Eastern Asia that is of local importance in Southwestern China and which might be a biotechnologically relevant resource in the future. The *Allium wallichii* microbiome was characterized by a high degree of microhabitat specificity especially between the aboveground and belowground parts, a missing rhizosphere effect, an unusual evenness within the bacterial community structure, and *Pseudomonas*-rich hotspots. *Pseudomonas* was identified as a biomarker for healthy onions [26], but is also a model for beneficial, plant-associated bacteria for a long time [30, 31]. Further exploration of the natural *Pseudomonas* population associated with Himalayan onion is a promising strategy to discover new biological resources for modern agriculture. It might harbor distinct strains that are transferable to its cultivated relatives that are often affected by disease outbreaks. Here, especially the high prevalence of soil-borne phytopathogenic fungi affects the cultivation of onion [32] and thus might profit from new biological solutions.

When the two endosphere habitats (endorhiza and pedicule endosphere) of the plant were compared, their bacterial community compositions showed substantial differences. The endorhiza showed a prevalence of *Actinobacteria* and *Enterobacteriaceae* at higher taxonomic levels while the pedicule endosphere was colonized by a high number of *Firmicutes*. Each of these bacterial groups showed a contrary trend in the respective other endosphere of *Allium wallichii*. We assume that this distribution is due to differences in physicochemical conditions of the two niches. Nevertheless, *Actinobacteria* as well as *Firmicutes* might harbor members that are of potential interest as new bioresources due to their high biocontrol potential [17, 33]. Interestingly, we have also observed a higher similarity of the bacterial community in the endorhiza to the community found on the plant’s flowers when compared with the rhizosphere microbiome. This is an indication for the high selectivity of the plant and a filter effect that was previously also observed with crop plants [11]. Soil-borne microorganisms have to either pass through the plant’s endosphere to reach aboveground plant parts or they are transferred by repelling raindrops or splashing irrigation water in cultivated plants [34].

We have identified two specificities in the *A. wallichii* microbiome that differentiates it from most other plants that were subjected to bacterial community analyses in the past years. First, the rhizosphere effect of the plants was low, and no significant differences were observed when compared with the surrounding soil. The complementary physicochemical soil analyses indicated that the characteristics of the natural monoculture are representative for this region. Previous studies that were conducted in the proximity of Jiucaiping Mountain reported pH values as well as total nitrogen concentrations that were in the same range [35, 36]. Plants direct a substantial amount of fixed nitrogen and carbon to the rhizosphere trough specific exudates [37]. These exudates attract and nourish populations of beneficial rhizosphere microorganisms through the plant’s whole lifecycle [20, 38]. We hypothesize that either the soil in the *A. wallichii* fields of Jiucaiping Mountain is influenced by the naturally occurring monoculture to a level that led to a homogenization of the rhizosphere and the surrounding soil, or that the plant has a low potential to attract microorganisms from the surrounding soil. The latter might be likely, because *A. wallichii* has a proportionally small root system that consists of a low number of primary roots. Plant exudates are mainly excreted from lateral roots, which also serve as entry points for nodulating bacteria or other beneficial endophytes [37, 39]. Further indications for the second hypothesis are given by the fact the bulb plants have a specific lifecycle and are often associated with a mycorrhiza [25]. Interestingly, distinct *Pseudomonas* strains are known due to their positive interactions with certain fungi as “mycorrhiza helper bacteria” [40]. Their high occurrence might be indicative for the same role in the *A. wallichii* microbiome, which remains to be confirmed. It is also noteworthy to mention that plants were collected during the flowering stage for the current study and that it was previously found that plants show a lower rhizosphere effect at later development stages [41, 42]. Targeted analyses will be required in the future to clarify if this also applies to members of the Amaryllidaceae plant family.

As a second specificity of the *A. wallichii* microbiome, our dataset was characterized by a high microbial diversity, and more remarkably by an exceptional evenness in the distribution of different taxa. This is indicative for a stable and undisturbed ecosystem and is not commonly observed with cultivated plants. While less attention is paid to community evenness than to diversity and microbial abundance in general [43], it is an important determinant for resilience of various ecosystems, especially under stress conditions [44]. In previous studies, a generally lower evenness was observed for plant microbiomes [14, 45], even if amendments that can increase the microbial community evenness were used [46]. We hypothesize that the observed evenness of the *A. wallichii* microbiome mainly results from the specific environmental conditions on the mountain plateau in combination with the large natural monoculture that has facilitated co-adaption of microbial communities with their plant host. Therefore, *A. wallichii* is also potentially interesting as a model plant to study the development and maintenance of evenly distributed bacterial communities for higher resilience against biotic as well as abiotic stress factors.

## Conclusions

In the present study, we could show that *A. wallichii* harbors a spatially structured microbiome that showed specific signatures in each tissue type. *A. wallichii* showed special characteristics in its microbiome that make it distinguishable from other plants that were subjected to analogous analyses in the past. Furthermore, the bacterial genus *Pseudomonas* was found to be a universal component of the plant’s bacterial communities. This makes it a promising candidate for targeted isolation and cultivation experiments in the future. In summary, the obtained insights provide a reference for microbiome studies within the Amaryllidaceae plant family as well as a basis for future approaches to mine for specific bioresources in the plant’s microbiota that are potentially exploitable as natural protectants of its cultivated relatives.

## Supplementary Information

ESM 1 (XLSX 1806 kb)

ESM 2 (DOCX 1083 kb)

## Data Availability

The 16S rRNA gene fragment amplicon sequencing data used in this study was deposited at the European Nucleotide Archive (ENA; https://www.ebi.ac.uk/ena) under the accession number PRJEB34098.

## Abbreviations

NaCl: Sodium chloride
rpm: Revolutions per minute
min: Minute
ml: Milliliter
cm: Centimeter
NaClO: Sodium hypochlorite
ddH_2_O: Double distilled water
DNA: Deoxyribonucleic acid
PCR: Polymerase chain reaction
PNA: Peptide nucleic acid
mPNA: Mitochondrial peptide nucleic acid
pPNA: Plastidial peptide nucleic acid
bp: Base pair
PCoA: Principal coordinate analysis
ANOSIM: Analyses of similarity
OTU: Operational taxonomic unit
FDR: False discovery rate
ANOVA: Analysis of variance
g: Gram
pH: Hydrogen ion concentration
NCBI: National Center for Biotechnology
*H*′: Shannon index
*E*: Evenness
kg: Kilogram
mg: Milligram
