# Supplementary material for: The Himalayan Onion (Allium wallichii Kunth) Harbors Unique Spatially Organized Bacterial Communities
Source: Microb Ecol. 2021 Mar 16;82(4):909–18. doi: 10.1007/s00248-021-01728-5 (PMC8551121; doi:10.1007/s00248-021-01728-5)
Supplement: Supplementary file 2 — (DOCX 1083 kb) [file 248_2021_1728_MOESM2_ESM.docx]

**Supplementary Material**

**The Himalayan onion (*Allium wallichii* Kunth) harbors unique spatially organized bacterial communities**

Xiaoyulong Chen^1,2,4^, Lisa Krug^3^, Maofa Yang^2^, Gabriele Berg^3^, and Tomislav Cernava ^2,3*^

^1^ Key Laboratory of Green Pesticide and Agricultural Bioengineering, Ministry of Education, Guizhou University, 550025, Guiyang, China

^2^ College of Tobacco Science, Guizhou University, 550025, Guiyang, China

^3^ Institute of Environmental Biotechnology, Graz University of Technology, 8010, Graz, Austria

^4^ College of Science, Tibet University, 850012, Lhasa, China

***Corresponding author:**

Tomislav Cernava - email: tomislav.cernava@tugraz.at, telephone: +43 316 8738312

**Submitted to**: Microbial Ecology

**Running title:** *Allium wallichii* microbiome

**Key words:** plant microbiome, bacterial communities, endophytic bacteria, rhizosphere, phyllosphere

**Table S2.** Significant differences in alpha diversity were assessed through pairwise comparison of Shannon indices using Kruskal-Wallis including Benjamini/Hochberg FDR correction. Significant differences (*p* < 0.05) are highlighted in *bold*.

| **Shannon – Kruskal-Wallis** | |  |
| --- | --- | --- |
| **Groups** | **p-value** | **q-value** |
| Endorhiza, Flower | 0.337 | 0.374 |
| Endorhiza, Pedicel | 0.144 | 0.180 |
| **Endorhiza, Rhizosphere** | **0.004** | **0.020** |
| **Endorhiza, Soil** | **0.011** | **0.021** |
| Flower, Pedicel | 0.584 | 0.584 |
| **Flower, Rhizosphere** | **0.004** | **0.020** |
| **Flower, Soil** | **0.011** | **0.021** |
| **Pedicel, Rhizosphere** | **0.006** | **0.021** |
| **Pedicel, Soil** | **0.014** | **0.024** |
| Rhizoshpere, Soil | 0.055 | 0.079 |

**Table S3**. Significant differences in alpha diversity were assessed through pairwise comparison of number of observed OTUs using Kruskal-Wallis including Benjamini/Hochberg FDR correction. Significant differences (*p* < 0.05) are highlighted in *bold*.

| **Observed otus – Kruskal-Wallis** | |  |
| --- | --- | --- |
| **Groups** | ***p*-value** | **q-value** |
| Endorhiza, Flower | 0.150 | 0.187 |
| Endorhiza, Pedicel | 0.201 | 0.224 |
| **Endorhiza, Rhizosphere** | **0.004** | **0.020** |
| **Endorhiza, Soil** | **0.011** | **0.021** |
| Flower, Pedicel | 0.584 | 0.584 |
| **Flower, Rhizosphere** | **0.004** | **0.020** |
| **Flower, Soil** | **0.011** | **0.021** |
| **Pedicel, Rhizosphere** | **0.006** | **0.021** |
| **Pedicel, Soil** | **0.014** | **0.024** |
| **Rhizoshpere, Soil** | **0.033** | **0.047** |

**Table S4.** Differences in community composition between different microhabitats associated with *A. wallichii* were calculating using anosim. Calculation is based on unweighted UniFrac distance matrices. Significant differences (*p* < 0.05) are highlighted in *bold*.

| **Unweighted UniFrac - anosim** | | |
| --- | --- | --- |
| **Groups** | **R** | ***p*-value** |
| Endorhiza, Flower | 0.072 | 0.297 |
| Endorhiza, Pedicel | 0.226 | 0.066 |
| **Endorhiza, Rhizosphere** | **1.000** | **0.010** |
| **Endorhiza, Soil** | **1.000** | **0.013** |
| Flower, Pedicel | 0.081 | 0.196 |
| **Flower, Rhizosphere** | **1.000** | **0.010** |
| **Flower, Soil** | **1.000** | **0.013** |
| **Pedicel, Rhizosphere** | **1.000** | **0.010** |
| **Pedicel, Soil** | **1.000** | **0.012** |
| Rhizoshpere, Soil | 0.194 | 0.099 |

**Table S5.** Significant differences in evenness were calculated by pairwise comparison using ANOVA including Banferroni multiple test correction. Significant differences (*p* < 0.05) are highlighted in *bold*.

| **Evenness - ANOVA** |  |
| --- | --- |
| **Groups** | ***p*-value** |
| Endorhiza, Flower | 1.000 |
| Endorhiza, Pedicel | 1.000 |
| **Endorhiza, Rhizosphere** | **0.031** |
| **Endorhiza, Soil** | **0.018** |
| Flower, Pedicel | 1.000 |
| Flower, Rhizosphere | 0.120 |
| Flower, Soil | 0.060 |
| Pedicel, Rhizosphere | 1.000 |
| Pedicel, Soil | 0.565 |
| Rhizoshpere, Soil | 1.000 |

**Table S6.** Prevalent bacterial taxa in the microbiome dataset. All taxa with a relative abundance lower than 0.5% were summarized as “other”.

| **Abundance (%) in specified sample type** | | | | | **Taxonomic assignments at different levels** | | | | |
| --- | --- | --- | --- | --- | --- | --- | --- | --- | --- |
| **Flower** | **Pedicel** | **Endorhiza** | **Rhizosph.** | **Soil** | **Phylum** | **Class** | **Order** | **Family** | **Genus** |
| 0.08 | 0.00 | 0.17 | 2.74 | 2.79 | Acidobacteria | Acidobacteria | Acidobacteriales | Acidobacteriaceae | f_Acidobacteriaceae |
| 0.04 | 0.00 | 0.08 | 1.74 | 2.91 | Acidobacteria | p_Acidobacteria | p_Acidobacteria | p_Acidobacteria | p_Acidobacteria |
| 0.00 | 0.00 | 0.04 | 1.67 | 1.68 | Acidobacteria | Solibacteres | Solibacterales | Solibacteraceae (Subgroup 3) | Bryobacter |
| 0.08 | 0.00 | 0.12 | 1.33 | 1.77 | Acidobacteria | Solibacteres | Solibacterales | Solibacteraceae (Subgroup 3) | Cand. Solibacter |
| 0.08 | 0.04 | 0.04 | 1.67 | 1.74 | Bacteroidetes | Sphingobacteriia | Sphingobacteriales | Chitinophagaceae | f_Chitinophagaceae |
| 0.08 | 0.00 | 0.29 | 1.15 | 2.08 | Bacteroidetes | Sphingobacteriia | Sphingobacteriales | Sphingobacteriaceae | Mucilaginibacter |
| 0.83 | 0.84 | 0.29 | 1.79 | 0.14 | Bacteroidetes | Bacteroidia | Bacteroidales | o_Bacteroidales | o_Bacteroidales |
| 0.83 | 2.44 | 0.37 | 0.49 | 0.31 | Bacteroidetes | Bacteroidia | Bacteroidales | Bacteroidaceae | Bacteroides |
| 2.62 | 0.34 | 0.08 | 0.51 | 0.03 | Bacteroidetes | Cytophagia | Cytophagales | Cytophagaceae | Hymenobacter |
| 0.04 | 0.00 | 0.04 | 1.18 | 0.85 | Bacteroidetes | Cytophagia | Cytophagales | Cytophagaceae | f_Cytophagaceae |
| 0.83 | 7.86 | 0.17 | 0.03 | 0.00 | Firmicutes | Clostridia | Clostridiales | Ruminococcaceae | Faecalibacterium |
| 1.23 | 1.51 | 0.50 | 1.00 | 0.34 | Firmicutes | Bacilli | Lactobacillales | Lactobacillaceae | Lactobacillus |
| 0.87 | 3.57 | 0.37 | 0.20 | 0.00 | Firmicutes | Bacilli | Lactobacillales | Streptococcaceae | Streptococcus |
| 0.75 | 0.71 | 1.24 | 0.05 | 1.03 | Firmicutes | Bacilli | Bacillales | Staphylococcaceae | Staphylococcus |
| 0.04 | 0.04 | 0.29 | 0.87 | 1.00 | Proteobacteria | Alphaproteobacteria | Rhizobiales | Bradyrhizobiaceae | Bradyrhizobium |
| 0.52 | 0.38 | 0.04 | 1.69 | 0.20 | Proteobacteria | Alphaproteobacteria | Rhizobiales | Methylobacteriaceae | Methylobacterium |
| 7.07 | 2.61 | 0.54 | 1.87 | 0.48 | Proteobacteria | Alphaproteobacteria | Sphingomonadales | Sphingomonadaceae | Sphingomonas |
| 1.15 | 0.92 | 0.99 | 2.49 | 2.05 | Proteobacteria | Betaproteobacteria | Burkholderiales | Comamonadaceae | f_Comamonadaceae |
| 1.83 | 0.59 | 0.12 | 0.20 | 0.31 | Proteobacteria | Betaproteobacteria | Burkholderiales | Oxalobacteraceae | Massilia |
| 0.64 | 0.46 | 7.11 | 0.67 | 1.08 | Proteobacteria | Gammaproteobacteria | Enterobacteriales | Enterobacteriaceae | f_Enterobacteriaceae |
| 1.19 | 0.55 | 1.24 | 0.00 | 0.00 | Proteobacteria | Gammaproteobacteria | Pseudomonadales | Moraxellaceae | Moraxella |
| 6.43 | 3.53 | 1.86 | 0.79 | 0.63 | Proteobacteria | Gammaproteobacteria | Pseudomonadales | Pseudomonadaceae | Pseudomonas |
| 0.04 | 0.00 | 0.17 | 1.43 | 0.91 | Proteobacteria | Gammaproteobacteria | Xanthomonadales | Xanthomonadales Inc. Sedis | Acidibacter |
| 0.08 | 0.00 | 5.33 | 0.15 | 0.26 | Actinobacteria | Actinobacteria | Corynebacteriales | Mycobacteriaceae | Mycobacterium |
| 0.00 | 0.00 | 0.00 | 1.00 | 1.28 | Chloroflexi | Ktedonobacteria | Ktedonobacterales | o_Ktedonobacterales | o_Ktedonobacterales |
| 0.04 | 0.00 | 0.04 | 2.36 | 3.42 | Planctomycetes | Planctomycetacia | Planctomycetales | Planctomycetaceae | f_Planctomycetaceae |
| 0.00 | 0.00 | 0.00 | 0.92 | 1.34 | Verrucomicrobia | p_Verrucomicrobia | p_Verrucomicrobia | p_Verrucomicrobia | p_Verrucomicrobia |
| 30.81 | 27.31 | 34.97 | 1.87 | 0.77 | __ | __ | __ | __ | unknown Bacteria |
| 7.86 | 8.19 | 11.37 | 0.00 | 0.00 | __ | __ | __ | __ | unassigned |
| 33.94 | 38.11 | 32.12 | 68.13 | 70.61 | other | other | other | other | other |

**
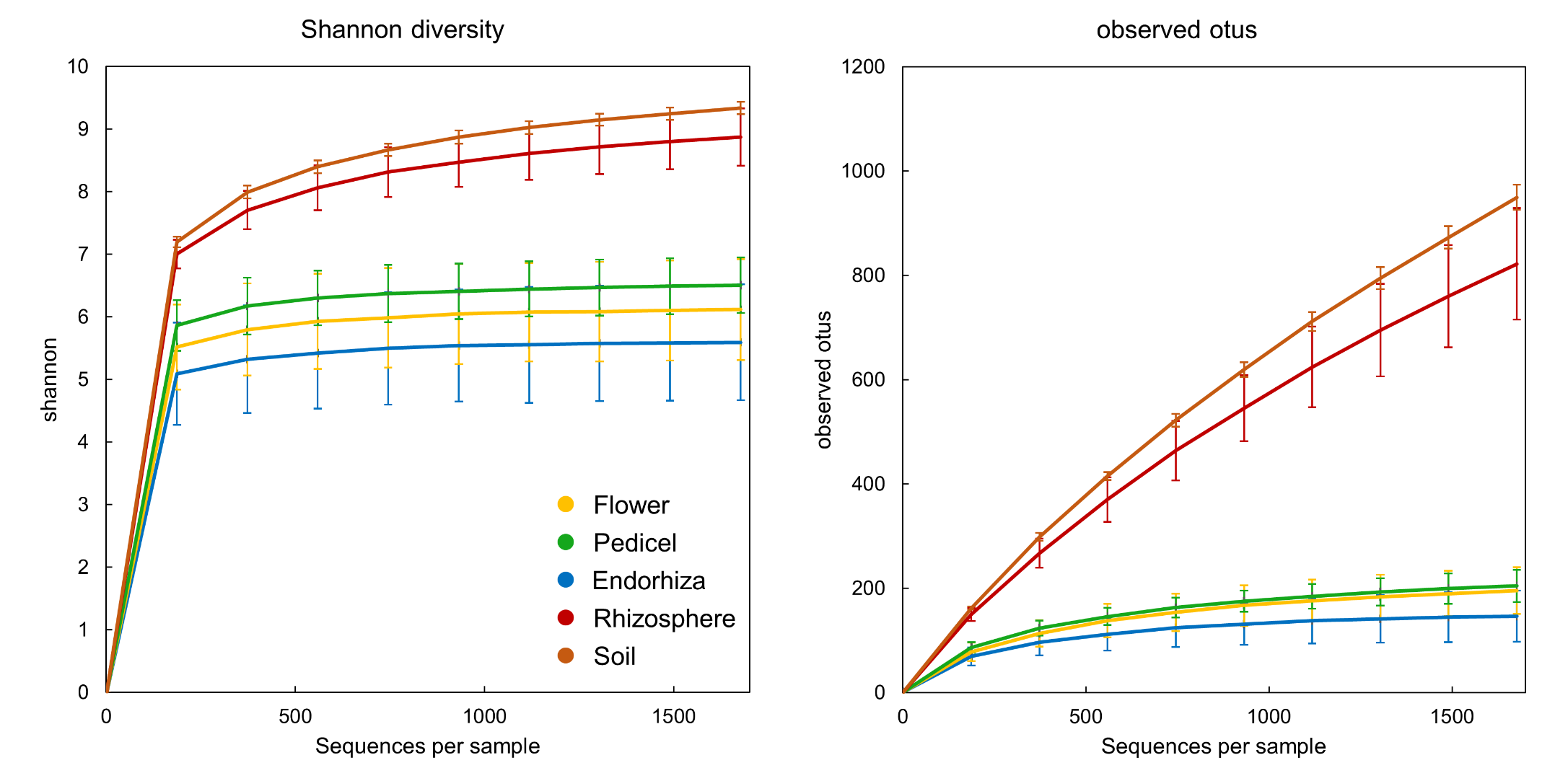
**

**Figure S1.** Diversity assessment based on Shannon index and Observed OTUs. Each color indicates a specific sample type. The standard error is indicated with error bars in each of the graphs.


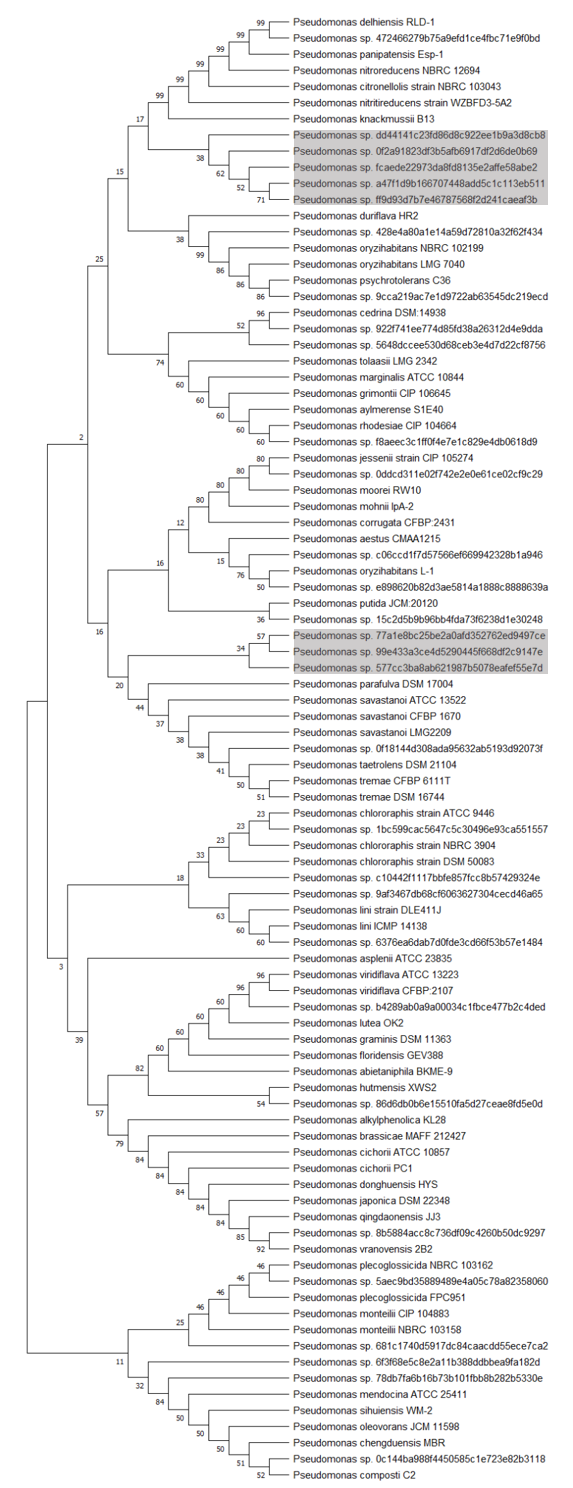


**Figure S2.** Phylogenetic tree with all *Pseudomonas* ASVs that were recovered from the 16S rRNA gene fragment library and reference strains (NCBI database). Feature IDs were included for each ASV. Two clusters with potentially new Pseudomonas lineages were highlighted in grey.
